# Evidence of health inequity in child survival: spatial and Bayesian network analyses of stillbirth rates in 194 countries

**DOI:** 10.1038/s41598-019-56326-w

**Published:** 2019-12-24

**Authors:** Daniel Adedayo Adeyinka, Babayemi Oluwaseun Olakunde, Nazeem Muhajarine

**Affiliations:** 1 Department of Community Health and Epidemiology, College of Medicine, University of Saskatchewan, Saskatoon, SK S7N 5E5 Canada; 2 Department of Public Health, Federal Ministry of Health, Abuja, Nigeria; 3 Department of Environmental and Occupational Health, School of Public Health, University of Nevada, Las Vegas, NV USA; 4 Saskatchewan Population Health and Evaluation Research Unit, Saskatchewan, SK S7N 2Z4 Canada

**Keywords:** Paediatric research, Paediatric research

## Abstract

Estimated at 2.6 million annually, stillbirths worldwide have stayed alarmingly high, in contrast to neonatal and under-five mortality rates. It is a neglected public health challenge globally, with less attention to its social determinants. We examined spatial patterns of country-level stillbirth rates and determined the influence of social determinants of health on spatial patterns of stillbirth rates. We also estimated probabilistic relationships between stillbirth rates and significant determinants from the spatial analysis. Using country-level aggregated data from the United Nations databases, it employed ecological spatial analysis and artificial intelligence modeling based on Bayesian network among 194 World Health Organization member countries. From the spatial analysis, thirty-seven countries formed a cluster of high values (hot-spots) for stillbirth and 13 countries formed a cluster of low values (cold-spots). In the multivariate regression, gender inequality and anaemia in pregnancy were significantly associated with spatial patterns of higher stillbirth rates, while higher antenatal care (ANC) coverage and skilled birth attendants during delivery were associated with clusters of lower stillbirth rates. The Bayesian network model suggests strong dependencies between stillbirth rate and gender inequality index, geographic regions and skilled birth attendants during delivery. The Bayesian network predicted that the probability of low stillbirth rate increased from 56% to 100% when the percentage of countries with high skilled birth attendants during delivery increased from 70% to 88%, high ANC coverage increased from 55% to 70%, high prevalence of anaemia in pregnancy decreased from 27% to 11% and high gender inequality index decreased from 43% to 21%. Recognizing the urgency in reducing stillbirths globally, multi-pronged strategies should be designed to promote gender equality and strengthen the reproductive and maternal health services in Africa, Eastern Mediterranean, South Eastern Asia, and other countries with disproportionately high stillbirth rates.

## Introduction

For international comparison, stillbirth is typically defined as foetal death weighing ≥1000 g or occurring at ≥28 weeks^1,2^. Estimated at 3 million annually^3,4^, stillbirths worldwide have stayed alarmingly high, in contrast to neonatal and under-five mortality rates^1^. Despite the repeated demands to improve child survival in low-and middle-income countries (LMICs) and among the marginalized population groups in high-income countries, stillbirth—an important public health challenge—is often neglected. This is evident by its consistent omission in the Millennium Development Goals (MDG) and Sustainable Development Goals (SDG)^5^. A major problem which arises in this domain of child health is the varying definitions of stillbirth, which makes policy reforms, global and national tracking challenging^6^. Attention has then focused on standardizing the definition and refining stillbirth rate estimates^7^. Increasing global awareness about the importance of continuum of care from conception to post-delivery phase led to the development of the “Every Newborn Action Plan” (ENAP) of the 2014 World Health Assembly—an effort aimed at reducing global stillbirth rate to 9 per 1000 total births and national stillbirth rate to 12 per 1000 total births (especially in the LMICs) by 2030^8^.

In recent systematic reviews by Christou *et al*.^9^ and Reinebrant *et al*.^10^, we observed that research to date has exclusively focused on the medical determinants of stillbirth, while its non-medical determinants have received little attention. Also, no study has quantified the relationship between stillbirth and its social determinants for evidence-based decision making. Hence, in most settings, Maternal, Newborn and Child Health (MNCH) programmes are often designed to address the medical causes of stillbirths such as intrapartum complications, post-term pregnancy, maternal infections in pregnancy (malaria, syphilis and HIV), maternal medical indicators (especially hypertension, hemorrhage, and diabetes), foetal growth restriction, and congenital abnormalities^1,11^. However, social determinants of health tend to be associated with maternal and child health outcomes and these have been underexamined in relation to stillbirths^12–14^.

In addition, there are widening social inequity gaps which occur along different dimensions such as geographical regions, socioeconomic positions, gender, and accessibility to and acceptability of health care services. Unlike health inequality, inequity refers to unjust and worse health outcomes among marginalized population which are often preventable^15^. Recently, some authors have enumerated the socioeconomic gains of implementing MNCH programmes within the wider context of population health for reducing stillbirths^16^. In order to remarkably reduce stillbirth rates, factors that place women at disadvantageous positions should therefore be addressed. As aggregated country-level data on stillbirth rates have been made available, there is an opportunity to explore the key social determinants of stillbirth across 194 countries, using ecological spatial modeling approach and Bayesian artificial intelligence to inform prevention efforts for achieving the ambitious targets of ENAP by 2030. The primary objective of this study was to examine spatial patterns of country-level stillbirth rates and determine the factors associated with the spatial disparity of stillbirth rate. The secondary objective was to estimate probabilistic relationships between stillbirth rates and significant determinants from the spatial analysis.

## Methods

### Study design and data sources

This is an ecological spatial and Bayesian network analyses that utilized country-level aggregated data which are publicly available from the United Nations (UN) databases (Supplementary Table S1). All the 194 World Health Organization (WHO) member countries^17^ were included in this study. According to WHO region categories, there were 46 African (AFR), 35 American (AMR), 22 Eastern Mediterranean (EMR), 53 European (EUR), 11 South East Asian (SEAR), and 27 Western Pacific (WPR) countries. The dependent variable—stillbirth rate was re-estimated by Blencowe and colleagues^18^ as the ratio of annual number of foetal deaths after 28 weeks to the total number of live births in a year^2^. The last estimation used for this study was conducted in 2015. The details of the calculations can be found at the WHO Global Health Observatory data repository website^2^.

There were 23 independent variables, being measured at the country-level and obtained from the World Bank^19^, United Nations Development Programme (UNDP)^20^, and WHO^21^. These independent variables were selected to represent the key aspects of the Social Determinants of Health (SDH) Framework^22^: socioeconomic and cultural factors, lifestyle, healthcare resources, maternal infections and conditions, and maternal and reproductive health service coverage indices.

**Figure 1:**
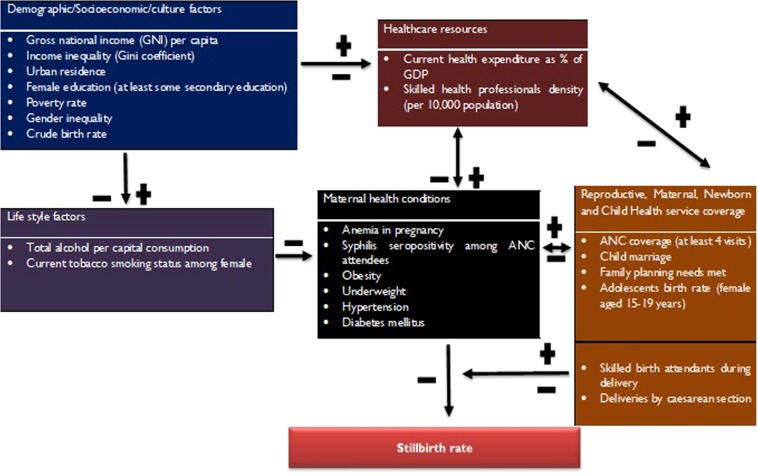
The conceptual model for the determinants of stillbirth (adapted from Social Determinants of Health Framework)^22^.

As shown in Fig. 1, the conceptual model hypothesized the different classes of variables influencing stillbirth along various pathways. At one end of the spectrum, the nations with good demographic and socioeconomic indices would have better healthcare system (including MNCH services) and health behaviour. The culture, social organization, and health behaviour would have a profound influence on maternal health conditions, which mediate stillbirth. Specifically, skilled birth attendants during delivery and delivery by caesarean section were proposed as potential modifiers/mediators for the relationship between maternal health conditions and stillbirth.

As shown in Supplementary Table S1, the national demographic, socioeconomic and cultural factors examined were Gross National Income (GNI) per capita, poverty rate, urban residence, female educational status, crude birth rate, gender inequality index, and income inequality. Gender inequality index (GII) measures gender disparity between men and women in the 3 dimensions; reproductive health, empowerment and labor market. The lowest possible score is zero (equality) and highest possible score is one (inequality)^20^.

For health behaviour/lifestyle, we used age-standardized prevalence of current smoking among adult females and total alcohol per capita consumption in adults. We also included current health expenditure as percentage of gross domestic product (GDP) and density of skilled health personnel (per 10 000 population) as proxies for healthcare resources. Maternal health conditions included prevalence of anaemia in pregnancy (adjusted for altitude and smoking), syphilis seropositivity among antenatal care (ANC) attendees, age-standardized prevalence of obesity among female adults, prevalence of underweight among female adults as a proxy for maternal nutrition, age-adjusted prevalence of hypertension among adult females and age-adjusted prevalence of diabetes mellitus in female adults. Indicators for healthcare service coverage included percentage of deliveries by caesarean section (measurement for access to emergency health care during childbirth), ANC coverage—at least 4 visits, skilled birth attendants during delivery, and proportion of women of reproductive age (married/in-union) who have their family planning needs satisfied with modern methods, adolescent birth rate and prevalence of child marriage—proxies for sexual and reproductive health. The reporting dates differ for independent variables, ranging from 2006 to 2017. GNI per capita, poverty rate, prevalence of current smoking among female adults, syphilis seropositivity, prevalence of underweight among female adults, density of skilled health personnel, and adolescent birth rates were observed to be skewed, hence they were log-transformed to ensure normality of the variables.

The philosophical perspectives for this study were drawn from postpositivist and transformative worldviews. The postpositivist view was employed to verify our theory and determine the relationship of key independent variables on stillbirth rate (i.e. deterministic and reductionistic lens). In addition, transformative perspective was used to raise the consciousness of stakeholders for political/programmatic changes to mitigate health disparities, especially in LMICs.

### Geospatial analysis

The spatial scale and unit of analysis is country. With GeoDa v. 1.12 software^23^, a distance based spatial weight matrix with a threshold distance of 5720 km was generated in a geographic coordinate shapefile obtained from ArcGIS^24^. This distance was the most appropriate after various calibrations with different distances; all the 194 countries were interlinked—a condition for determining spatial dependence^25^. Furthermore, the adequacy of spatial weights was confirmed with the symmetry of connectivity histogram and connectivity map^25^.

To examine spatial dependence, cluster analyses were performed. The global and local spatial autocorrelations were measured with Global Moran’s I index and Local Moran’s I index, respectively. Moran’s I index value ranges from +1 to −1, indicating strong positive autocorrelation (perfect clustering) to negative autocorrelation (perfect dispersion)^26^. Correspondingly, the positive and negative indices suggest aggregation of neighbourhoods with similar and different values across geographical space than the expected random distribution, while zero implies no autocorrelation (i.e. perfect randomness). The local indicator of spatial autocorrelation (LISA) cluster maps were generated to show hot- and cold-spots of statistically significant spatial clusters of neighbouring countries with high and low stillbirth rates, respectively. Randomizations were set at 999 Monte Carlo permutation to ensure adequate statistical power for p-value < 0.05. Following the proposed algorithm for spatial regression by Anselin^25^, the associations between stillbirth rate and the independent variables were initially examined with ordinary least squares (OLS) regression and tested for multicollinearity (condition index >30)^25^. For the purpose of minimizing data loss, pairwise deletion was utilized to treat missing data. Data imputation was not considered because of its tendency to underestimate standard errors and overestimate test statistic, hence producing biased results. Where the OLS model diagnostics (Lagrange multiplier lag and Lagrange multiplier error tests)^25^ indicated spatial dependence, the models were fitted with spatial lag or spatial error regression, as appropriate; otherwise OLS result was reported. Multiple block entries in multivariate spatial regression were performed using parsimonious backward approach. For the final multivariate model, we selected covariates that significantly predicted spatial pattern of stillbirth rate from the multiple regression models (models 1 to 5). The spatial regression model with the best goodness-of-fit was determined by R-squared, log likelihoods of the maximum likelihood estimations (MLE), Bayesian Information Criterion (BIC) and Akaike’s Information Criterion (AIC). To visually represent the key determinants of spatial pattern of stillbirth rates in high-dimensional geometry, a multivariate parallel coordinate plot was generated.

### Bayesian network analysis

As spatial models could only show the strengths of association but not interrelationships among the determinants studied, we further generated Bayesian network model with an artificial intelligence modeling and machine learning software—GeNIe v. 2.4.4 software^27^ to test our theories with the aggregated dataset for the 194 WHO countries. The network structure was generated based on the author’s programmatic knowledge of the temporal precedence of the variables. With significant level parameter set to p-value = 0.05 in PC structural learning algorithm, the variables were assigned as follows; tier 1 (region), tier 2 (gender inequality index), tier 3 (prevalence of anaemia in pregnancy and ANC coverage), tier 4 (skilled birth attendants during delivery), and tier 5 (stillbirth rate). The network structure was assessed to ensure that there was no residual confounding (through back-door path) and to show plausible paths among the significant variables from the final regression model. The network model learnt the parameters by using Expectation-Maximization algorithm to account for the missing values. The model accuracy was evaluated by using leave-one-out cross-validation method. The strengths of influence (corresponding contributions) for the variables were denoted by the thickness of Euclidean weighted and normalized arc widths. In addition, we assessed the direction of influence by using arc coloring in dynamic influence mode. The dynamic influence mode was used because it is context-specific and adjusts indirect influences, unlike static mode^28^. The green, red, grey and purple colors correspond to positive, negative, null and ambiguous influences of various factors on the probability distribution of stillbirth rate, respectively^28^. The aggregated, continuous data were grouped into categories based on either the median or mean values. The categorizations are as follows: stillbirth rate (high >12, low ≤12 per 1000 births); gender inequality index (high >0.4, low ≤0.4); ANC coverage (high >75%, low ≤75%); skilled birth attendants during delivery (high >85%, low ≤85%); and anaemia in pregnancy (high >40%, low ≤40%).

By using supervised machine learning, we generated prediction scenario that reflected the probability of each parameter, given that the proportion of countries with low stillbirth rates increased to 100% (desired state) from baseline for each WHO regions.

We performed sensitivity analysis to determine the extent to which stillbirth rate is accounted for by the explanatory variables. To validate the model calibration with the dataset, logarithmic loss, quadratic loss (Brier score) and spherical payoff were evaluated. Logarithmic loss values should be between zero and infinity, with zero indicating the best goodness-of-fit^29^. The quadratic loss value should lie between zero and two, where zero is the best performance^29^. Also, the spherical payoff should be between zero and one, with one indicating a perfect fit^29^. Similarly, the goodness-of-fit was assessed with area under receiver operating characteristics (ROC) and sensitivity tornado plot (Supplementary Fig. S1).

### Ethical approval

This study was exempted from ethical review by the full complement of the University of Saskatchewan Behavioural Ethics Committee (ID# 1066) because it relied on publicly available aggregated de-identified dataset.

## Results

**Figure 2:**
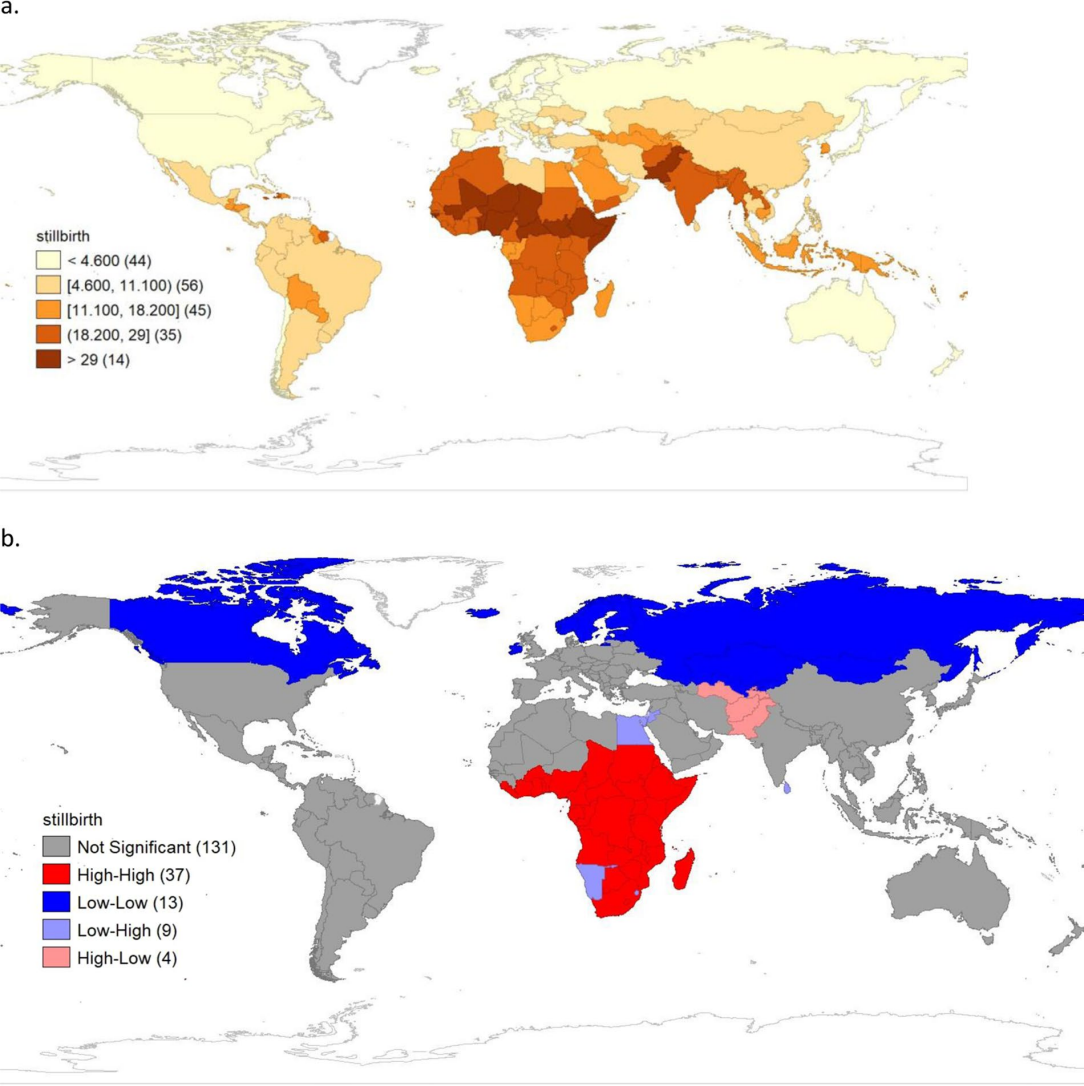
(**a**) Spatial distribution of stillbirth rates by country, 2015. (**b**) Univariate local indicators of spatial association (LISA) cluster map of stillbirth rate, 2015.

Table 1 presents the descriptive analysis of stillbirth rates and the independent variables. As shown in Fig. 2a, stillbirth rates varied considerably across the 194 countries. The average stillbirth rate was 12.8 (Standard Deviation, SD: 9.5) per 1000 total births, ranging from 1.3 per 1000 total births in Iceland to 43.1 per 1000 total births in Pakistan. The African region had the highest mean stillbirth rate, 23.0 (SD: 7.9) per 1000 total births, ranging from 9.5 per 1000 total births in Seychelles and Mauritius to 42.9 per 1000 total births in Nigeria. The lowest mean rate was observed in the European region, 5.0 (SD: 3.8) per 1000 total births, ranging from 1.3 per 1000 total births in Iceland to 17 per 1000 total births in Turkmenistan. With global Moran’s I index of 0.1 (p = 0.001), there was significant positive spatial autocorrelation (clustering) of stillbirth rates across the countries. The LISA cluster map (Fig. 2b) shows high-high clusters (neighbouring countries with significantly high stillbirth rates clustered to form hot-spots) involving 37 countries (19.1%). All the high-high clusters were in the WHO African region and Eastern Mediterranean region (Somalia, South Sudan, Sudan and Djibouti). The cold-cold clusters (cold-spots) were formed by clustering of 13 neighbouring countries (6.7%) with significantly low stillbirth rates. The cold-cold clusters were formed by Canada, Ireland, Estonia, Finland, Iceland, Kyrgyzstan, Kazakhstan, Latvia, Mongolia, Norway, Russia, Sweden, and Uzbekistan. Adjacent to these hot- and cold-spots were outliers—low-high and high-low clusters, respectively. The low-high clusters indicated 9 countries (4.6%) with significantly low stillbirth rates that were neighbours to countries with significantly high stillbirth rates (Sri Lanka, Egypt, Jordan, Mauritius, Maldives, Seychelles, Swaziland, Namibia, and Israel). Conversely, high-low clusters involved four countries: Afghanistan, Pakistan, Tajikistan, and Turkmenistan.

**Table 1:** Descriptive analysis of stillbirth rates and independent variables.

Variables	Mean (SD)/median (IQR)	Range	N (number of countries reporting)
**Dependent variable**			
Stillbirth rate (per 1000 total births)	12.8 (9.5)	1.3–43.1	194
**Independent variable Socio-economic/cultural factors**			
GNI per capita (Atlas method) US$	4970 (1880–14180)	290–80560	182
Income inequality (Gini coefficient)	38.5 (8.1)	16.6–63	157
Population living in urban areas (%)	56.0 (23.6)	9.1–100	190
Population of females with at least some secondary education (%)	61.2 (29.7)	1.7–100	159
Poverty rate (%)	3.0 (1.9–29.0)	1.9–87.7	115
Gender inequality index	0.4 (0.2)	0.04–0.8	156
Crude birth rate	21.7 (10.5)	6.8–49.7	190
**Lifestyle factors**			
Total alcohol per capita consumption	6.2 (4.1)	0.0–15.2	189
Prevalence of current tobacco smoking among female (%)	8.9 (3.1–19.9)	0.2–53.5	126
**Healthcare resources**			
Density of skilled health personnel (per 10 000 population)	45.7 (15.5–93.2)	1.1–271.6	178
Current health expenditure (%) of GDP	6.8 (3.0)	2.0–22.1	190
**Maternal infections and conditions**			
Prevalence of anaemia among pregnant women (%)	34.7 (11.1)	16.2–63.0	186
Syphilis seropositivity among pregnant women (%)	0.6 (0.1–1.9)	0.0–100.0	143
Prevalence of obesity among female adults (%)	23.4 (12.4)	2.6–63.3	190
Prevalence of underweight (BMI < 18 kg/m^2^) among female adults (≥18 years) (%)	3.4 (2.0–8.3)	0.3–24.2	188
Prevalence of hypertension among female adults (%)	22.4 (5.6)	8.2–35.8	190
Prevalence of diabetes mellitus among female adults (%)	9.8 (5.1)	2.8–28.4	189
**Maternal and reproductive health service coverage**			
Skilled birth attendants during delivery (%)	85.7 (20.4)	9.4–100.0	182
ANC coverage - at least four visits (%)	73.5 (21.4)	6.3–100.0	148
Adolescent birth rate (per 1000 women)	44.4 (13.5–80.0)	0.7–229.0	170
Prevalence of child marriage (%)	23.7 (15.0)	2.0–76.0	122
Deliveries by caesarean section (%)	20.2 (13.8)	0.9–58.1	171
Percentage of women of reproductive age who have their family planning needs satisfied (%)	55.1 (21.1)	5.6–89.8	118

SD- Standard Deviation; IQR- Interquartile range.

**Figure 3:**
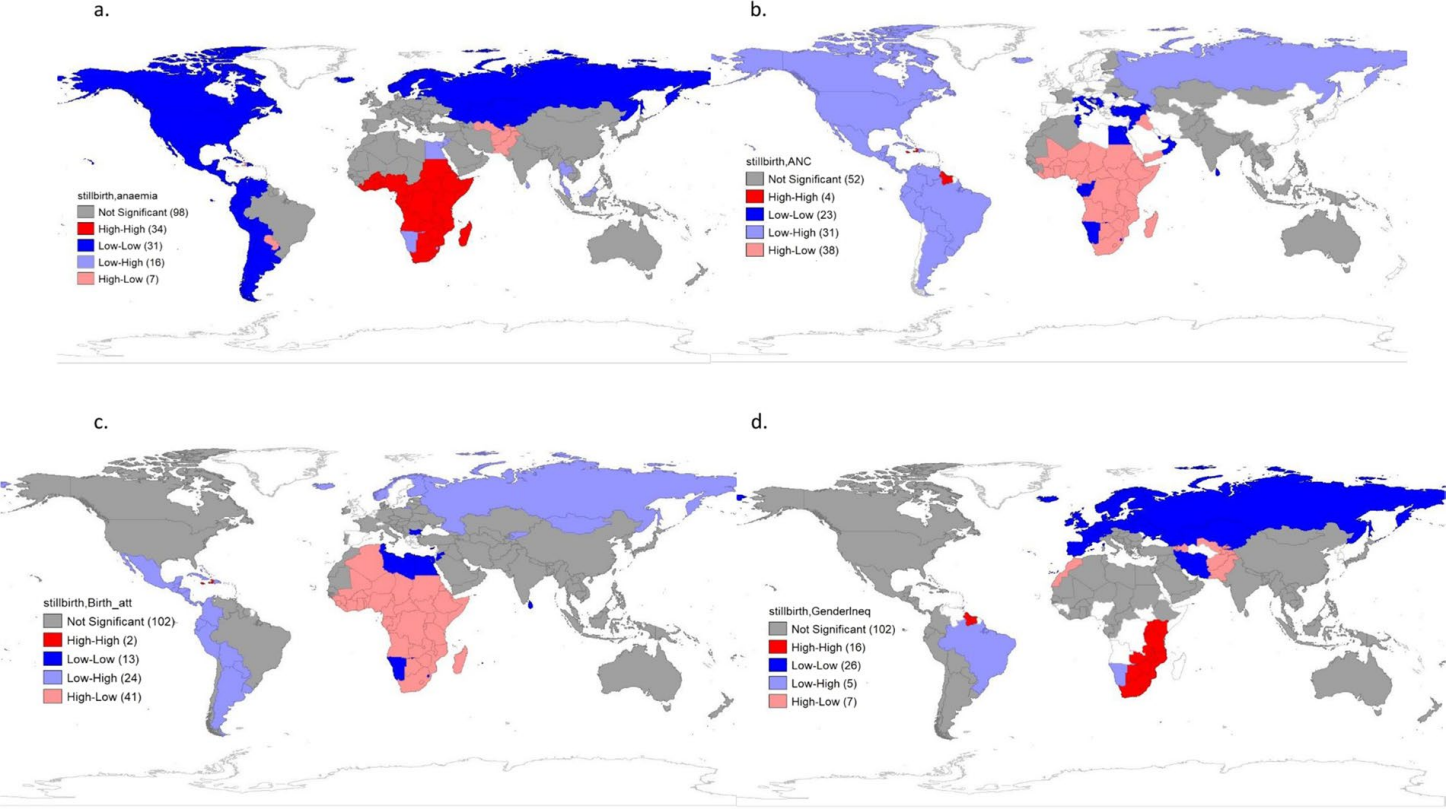
Adjusted local indicators of spatial association (LISA) cluster map of stillbirth rate and: (**a**) prevalence of anaemia in pregnancy, (**b**) ANC coverage, (**c**) Skilled birth attendants at delivery, (**d**) Gender inequality index.

The results of the bivariate spatial regression models are shown in Supplementary Table S2. The bivariate correlation between crude birth rate and poverty rate was high (r = 0.9). Also, gender inequality index was highly correlated with female education (r = −0.8), crude birth rate (r = 0.8), gross national income (GNI) per capita (r = −0.9), poverty rate (r = 0.8), and density of skilled health personnel (r = −0.9). Skilled birth attendants at delivery was highly correlated with poverty rate (r = −0.8) and density of skilled health personnel (r = 0.8). In the unadjusted models, stillbirth rate was significantly associated with all the independent variables except country-level prevalence of diabetes mellitus among female adults (ß = 0.11, p-value = 0.471). The best fitting (final) regression model included income inequality (Gini coefficient), gender inequality index, total alcohol consumption, tobacco consumption, anaemia in pregnancy, ANC coverage, and skilled birth attendants during delivery (Table 2). In the final model, the associations between stillbirth rate, and income inequality (ß = −0.04, p-value = 0.459), total alcohol consumption (ß = −0.27, p-value = 0.07), tobacco consumption (ß = −0.36, p-value = 0.405) became statistically not significant. After adjusting for the confounding effects of other covariates, the magnitude of coefficients decreased by 67.4% (from 42.0 to 13.7) for gender inequality index, 0.62 to 0.13 (79.0%) for anaemia in pregnancy, however, increased from −0.35 to −0.15 (57.1%) for ANC coverage and −0.36 to −0.12 (66.6%) for skilled birth attendants during delivery. While 1% increase in ANC coverage and skilled birth attendants during delivery reduce stillbirth rate by 0.2% and 0.1%, respectively; a unit rise in gender inequality index and 1% rise in prevalence of anaemia in pregnancy increase stillbirth rate by 13.7% and 0.1%, respectively. These seven variables explained 86% of the variance in stillbirth rate (adjusted R^2^ = 0.86). The adjusted LISA cluster maps (Fig. 3a–d) and multivariate parallel coordinate plot of the significant explanatory variables (not shown) also corroborate the findings from the final multivariate regression model.

**Table 2:** *Multivariate model of the determinants of stillbirth.

Variables	Model 1 β (p-value)	Model 2 β (p-value)	Model 3 β (p-value)	Model 4 β (p-value)	Model 5 β (p-value)	Final Model^≠^ β (p-value)
**Socioeconomic/culture**						
Urban residence	−0.03 (0.294)					
Income inequality	−0.21 (0.001)					−0.04 (0.459)
Poverty rate, log	2.48 (<0.001)					
Gender inequality index	31.86 (<0.001)					13.70 (0.01)
**Lifestyle**						
Total alcohol consumption		−0.60 (0.001)				−0.27 (0.07)
Tobacco consumption, log		−3.01 (<0.001)				−0.36 (0.405)
**Healthcare resources**						
Current health expenditure			−0.12 (0.401)			
Density of skilled health personnel, log			−6.58 (<0.001)			
**Maternal health conditions**						
Anaemia in pregnancy				0.28 (<0.001)		0.13 (0.03)
Obesity among female adults				0.12 (0.23)		
Hypertension among female adults				0.79 (<0.001)		
Syphilis seropositivity among pregnant women, log				0.59 (0.148)		
Prevalence of underweight among female adults, log				2.01 (0.214)		
**Health service coverage**						
Deliveries by caesarean section					−0.13 (0.001)	
ANC coverage - at least four visits					−0.21 (<0.001)	−0.15 (<0.001)
Skilled birth attendants during delivery					−0.16 (<0.001)	−0.12 (0.001)
Adjusted R^2^	0.79	0.41	0.66	0.66	0.77	0.86
Model	OLS	Spatial error	Spatial error	OLS	Spatial error	OLS

OLS- ordinary least square regression + Crude birth rate was dropped from the model because of its high multicollinearity with log poverty rate (r = 0.9). Also, gender inequality index was highly correlated with female education (r = −0.8), crude birth rate (r = 0.8) and log gross national income (GNI) per capita (r = −0.9).

^≠^Final model could not consider log poverty rate and log density of skilled health personnel because of multicollinearity issues with gender inequality index, log poverty rate (r = 0.8) and log density of skilled health personnel (r = −0.9). Skilled birth attendants at delivery was highly correlated with log poverty rate (r = −0.8) and log density of skilled health personnel (r = 0.8).

*Multiple block entries employed in building multivariate spatial regression models using parsimonious approach. Model 1 included: socioeconomic/culture (Urban population, income inequality, log of poverty rate, gender inequality index). Model 2: lifestyle (Total alcohol consumption, log of tobacco consumption). Model 3: Healthcare resources (Current health expenditure, log of skilled health personnel). Model 4: Maternal health conditions (Anaemia in pregnancy, obesity among female adults, hypertension among female adults, log of syphilis seropositivity among pregnant women, log of prevalence of underweight among female adults). Model 5: Health service coverage (Deliveries by caesarean section, ANC coverage, skilled birth attendants during delivery). Final Model: Income inequality, gender inequality index, total alcohol consumption, log of tobacco consumption, anaemia in pregnancy, ANC coverage, skilled birth attendants during delivery.

**Figure 4:**
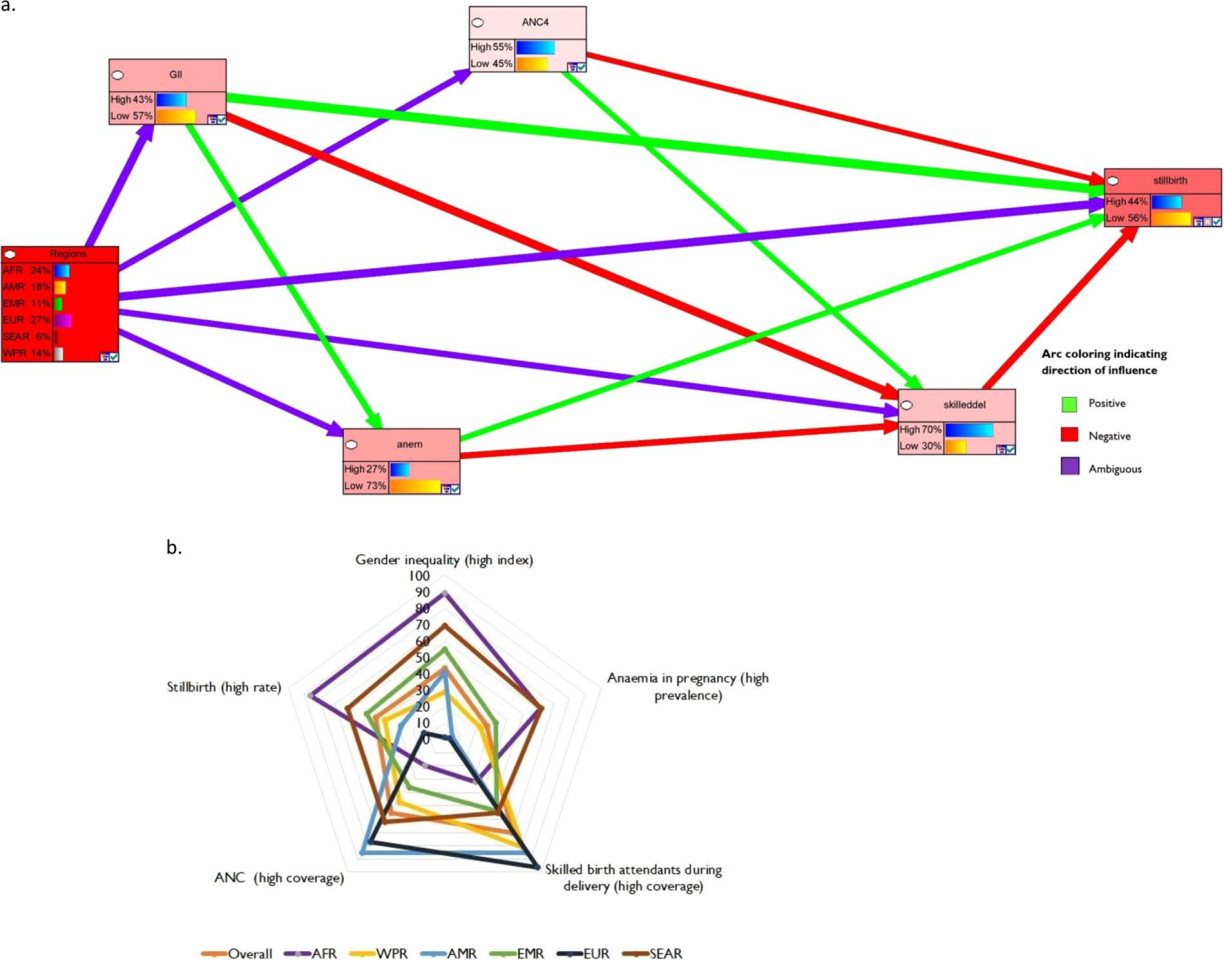
(**a**) Bayesian network of overall (status quo) probabilistic inference of WHO regions, gender inequality, ANC coverage, skilled birth attendants at delivery, anaemia in pregnancy on stillbirth rate. (**b**) Probabilistic inferences (%) of the key determinants for each WHO region. **Note**: (i) Strength of influence displayed by weighted normalized width of links. (ii) Green arcs represent strengthening effects, while red arcs and purple arcs represent weakening and unclear effects, respectively (iii) Stillbirth rate (high >12, low ≤12 per 1000 births); Gender inequality index (high >0.4, low ≤0.4); ANC coverage (high >75%, low ≤75%); skilled birth attendants during delivery (high >85%, low ≤85%); anaemia in pregnancy (high >40%, low ≤40%). (iii) WHO region: African (AFR), American (AMR), Eastern Mediterranean (EMR), European (EUR), South East Asian (SEAR), and Western Pacific (WPR).

From the Bayesian network model (Fig. 4a), gender inequality index had the strongest influence on stillbirth rate, with a weighted arc of 0.26, followed by geographic regions (0.24) and skilled birth attendants during delivery (0.21). As shown in Fig. 4a, the green arcs suggest that, high gender inequality index and high prevalence of anaemia in pregnancy increased the probability of high stillbirth rate. Also, red arcs show that, reduction in ANC coverage and skilled birth attendants during delivery increased the probability of stillbirth rates. However, the influence of region on stillbirth is shown to be ambiguous as depicted by the purple arc. Furthermore, the sensitivity tornado plot (Supplementary Fig. S1) depicts that the factors that influenced high stillbirth rates were: African region, high gender inequality index, low ANC coverage, low skilled birth attendants during deliveries and high prevalence of anaemia in pregnancy. Based on posterior probabilistic reasoning (Fig. 4b), Bayesian network suggests that African region had the highest probability of high stillbirth rate (86%); in addition, there were 89% probability of high gender inequality index, 61% probability of high prevalence of anaemia in pregnancy, 32% probability of high coverage for skilled birth attendants during delivery, and 20% probability of high ANC coverage. Similar patterns were observed for Eastern Mediterranean and South East Asian regions (Fig. 4b).

The Supplementary Table S3 shows the acceleration scenario for the individual regions. In the acceleration scenario (assuming no country has high stillbirth rate), the Bayesian network analysis shows that the probability of achieving a desired state, i.e. low stillbirth rate increased from 56% to 100%, occurred when the percentage of countries with high skilled birth attendants during delivery increased by 25.7% (from 70% to 88%), high ANC coverage increased by 27.3% (from 55% to 70%); and high prevalence of anaemia in pregnancy decreased by 59.3% (from 27% to 11%) and high gender inequality index decreased by 51.2% (from 43% to 21%).

The Bayesian network model had both very good fit and prediction accuracy (logarithmic loss, quadratic loss and spherical payoff were 0.3, 0.2 and 0.9, respectively). In addition, the area under receiver operating characteristic (ROC) of 0.84 was observed, translating to sensitivity of 77%, specificity of 80%, positive predictive value of 74%, negative predictive value of 82%, and accuracy rate of 78.9%.

## Discussion

Globally, distribution of stillbirth rates in relation with social determinants of health is not fully understood. Our study reveals spatial disparity in stillbirth rates and highlights the regions most affected (Africa, Eastern Mediterranean and South East Asia) as depicted by the cluster maps. In addition, we explored the relationship between social determinants of stillbirth with spatial regression and Bayesian network. Increased stillbirth rate was significantly associated with spatial patterns of increased gender inequality index and anaemia in pregnancy. In contrast, low stillbirth rate was significantly associated with spatial patterns of increased percentage of skilled birth attendants during delivery and ANC coverage. Stillbirths continue to be a global phenomenon, partly due to the inequitable social circumstance in which people live. Although there is scanty evidence on how social injustice fuel stillbirth, Evans *et al*. argued that social systems hugely contribute to poor health among the deprived population^30^.

This study observed mean stillbirth rate of 12.8 per 1000 total live births. However, huge variation exists at regional and country-levels. Figure 2a,b elucidate that African, Eastern Mediterranean, and South East Asian countries are being left behind, as many of the areas were hot-spots for stillbirth (countries with high rates compared to neighbouring countries). Also, further improvements are needed in other regions as they are disproportionately affected. For example, rates in the American region vary from 3.0 (United States of America) to 24.9 per 1000 total births in Haiti.

Consistent with previous studies^31–35^, our analysis underscores the importance of women’s autonomy in the society. This study suggests that gender inequality is the most important social determinants of stillbirth, as evident by its highest coefficients from the unadjusted and adjusted regression models. Also, the Bayesian network establishes that gender inequality index is the strongest influencer of stillbirth.

Geographically focusing on the elimination of gender inequality is crucial not only because it is a well-studied barrier to effective reproductive, maternal and child health services utilization, but our study suggests very high correlation between gender inequality index, and gross national income per capita, female education, poverty rate and crude birth rate. This buttresses the notion that gender inequality is an immensely important determinant of stillbirth that drives socioeconomic development. Countries should therefore ensure equitable polices to improve social interventions such as girls’ education and social assistance^36,37^. Researchers have demonstrated that smart investments in empowerment of women through education is central to achieving global health goals (especially SDG)^31–35^. Relatedly, addressing gender inequality provides opportunities for pregnant women to embrace child spacing and meet the high nutritional demands^38,39^ (especially iron) at third trimester, which may not be sufficiently provided in their diets^40–42^. Although nutritional anaemia arising from iron-deficiency is the most common cause of anaemia in pregnancy globally, women in tropical climate such as Sub-Saharan Africa might have anaemia due to malaria or parasitic infestations^43^. Overall, such variations in the association between anaemia in pregnancy and stillbirth rate reflect the level of national ANC coverage. Most of the pregnancy-related conditions are likely to be prevented or detected and treated during ANC visits. This study indicates markedly low stillbirth rates among countries with high ANC coverage and skilled birth attendants during deliveries. This result ties well with previous studies that reflect similar patterns of stillbirth rate with ANC coverage and skilled birth attendants during delivery^4,5^. The quality of ANC care and intrapartum care are therefore vital in improving child survival. It is on this note that WHO in 2016, recommended minimum of eight contacts of ANC visits^44^; however, this is yet to be fully implemented in many countries and not being tracked globally.

As observed from this study, gender inequality could be used as a proxy measure of poverty rate because they were highly correlated (r = 0.8). High poverty rate might have serious consequences on child survival. More meaningful poverty reduction approaches should focus on indigent individuals within countries, rather than directing international financial aids to poor countries without any target population in mind. This will go a long way to financially support the most vulnerable populations, hence reducing the social deprivation gap within countries. Indeed, it is paramount for LMICs to develop national policies that will strengthen their institutional structures especially for shortage of skilled health personnel. Contrary to the general assumptions of social determinants of health, we did not find significant associations between stillbirth rate, and urban residence, lifestyle factors (alcohol and tobacco consumption) and income disparity. With exception to urban residence, a similar conclusion was reached from an ecological cross sectional study of 20 Latin American countries^45^. Furthermore, our study did not observe significant association between diabetes among female adults and stillbirth. The loss of statistical power observed could be due to ecological nature of our analysis and inability to disaggregate the timing of diabetes mellitus into gestational diabetes and pre-existing diabetes. In a population-based cohort study among 92 218 singletons in England, pre-existing diabetes mellitus was observed to be a risk factor for stillbirth, however, no relationship could be established for gestational diabetes^46^. The authors noted that gestational diabetes was much likely to be well-controlled than pre-existing diabetes^46^.

This study has some unique strengths. To our knowledge, there have been no studies on spatial analysis and Bayesian network analysis that addressed social determinants of stillbirth. This study applied spatial cluster analysis and spatial regression modeling to understand differences in stillbirth rates among high- and low-performing countries (i.e. cold-spots and hot-spots, respectively) using the most recent aggregated data from the UN-agencies. Also, it examined inequities in a range of social determinants of health across the 194 WHO-member countries. The global approach of determining intercountry spatial variation suggests generalizability of the study findings.

Furthermore, the Bayesian network was able to qualitatively and quantitatively test the theorical underpinnings of this study. Nevertheless, few potential limitations of the study design should be considered. Regarding the limitations of ecological studies, ecological fallacy is apparently inherent in this study. This implies that the observed association at the aggregated country-level might not reflect association at the individual-level. Also, cause-and-effect relationship cannot be directly inferred from our analysis; the determinants of health are not necessarily the aetiological causes of ill health^47^. Also, some factors utilized in this analysis (e.g. GNI per capital and gender inequality) were not concurrently measured with stillbirth. Furthermore, possible confounding factors such as female genital cutting, intimate partner violence and congenital abnormalities could not be considered, because they were not available for most countries.

## Conclusions and Policy Implications

The result of this study supports intra- and inter-regional disparity in stillbirth rates. Gender inequality, inadequate access to ANC, non-availability of skilled birth attendants during delivery and anaemia in pregnancy contributed to variation of stillbirth. The countries with high gender inequality (probably due to low maternal education and high poverty rate) were associated with high prevalence of anaemia in pregnancy, low ANC coverage and skilled birth attendants during deliveries, and more likely to have high stillbirth rate. The picture is worse in the African, Eastern Mediterranean, and South East Asian countries. A global, regional and national re-focus on the determinants of stillbirth is required, while attention to and mitigation of the drivers of social inequities must be the core strategy. From this standpoint, future qualitative/mixed method studies or realist evaluations are needed to comprehend the context at a more granular level, especially for the hot-spots and geographical outliers (low-high and high-low clusters). In addition, we recommend individual-level studies to identify the most deprived population within the countries.

Recognizing the urgency in achieving the “Every Newborn Action Plan” targets, multi-pronged strategies should be designed to promote gender equality and strengthen the reproductive and maternal health services in Africa, Eastern Mediterranean, South East Asia and other countries with disproportionately high stillbirth rates. This warrants multidisciplinary and multisectoral collaborations between national governments, development agencies, civil societies and research institutions.

## Supplementary information

SUPPLEMENTARY APPENDIX: Evidence of health inequity in child survival: spatial and Bayesian network analyses of stillbirth rates in 194 countries

## Data Availability

The datasets analyzed during the current study are available at United Nations-websites.
