# Supplementary material for: Evidence of health inequity in child survival: spatial and Bayesian network analyses of stillbirth rates in 194 countries
Source: Sci Rep. 2019 Dec 24;9:19755. doi: 10.1038/s41598-019-56326-w (PMC6930217; doi:10.1038/s41598-019-56326-w)
Supplement: Supplementary file 1 — SUPPLEMENTARY APPENDIX: Evidence of health inequity in child survival: spatial and Bayesian network analyses of stillbirth rates in 194 countries [file 41598_2019_56326_MOESM1_ESM.pdf]

SUPPLEMENTARY APPENDIX

Evidence of health inequity in child survival: spatial and Bayesian network analyses of stillbirth rates in 194 countries

*Daniel Adedayo Adeyinka (ORCID: 0000-0003-1855-6878)[1,2], Babayemi Oluwaseun Olakunde[3], Nazeem Muhajarine (ORCID: 0000-0001-6781-5421)[1,4]

[1]Department of Community Health and Epidemiology, College of Medicine, University of Saskatchewan, Saskatoon, SK S7N 5E5, Canada
[2]Department of Public Health, Federal Ministry of Health, Abuja, Nigeria
[3]Department of Environmental and Occupational Health, School of Public Health, University of Nevada, Las Vegas, NV, USA
[4]Saskatchewan Population Health and Evaluation Research Unit, Saskatchewan, SK S7N 2Z4 Canada

*Correspondence to: DA Adeyinka daa929@usask.ca

Table of contents

Table S1. Description of variables and data sources 2

Table S2: Bivariate spatial regression of the determinants of stillbirth 6

Table S3: Predicted scenarios of the inferential analysis based on joint probabilities of region and stillbirth 7

Figure S1: Sensitivity tornado plot for low stillbirth rate across WHO-regions, 2015 8

SUPPLEMENTARY TABLES

Table S1: Description of variables and data sources

Variable	Operational definition	Year	Data source	Number of reporting countries
**Dependent**				
Still birth (per 1000 total births)	Third trimester fetal deaths (≥1000 grams or ≥28 weeks)	2015	http://apps.who.int/gho/data/view.main.GSWCAH06v	194
**Independent**				
**Socio-economic/ cultural factors**				
GNI per capital (Atlas method)	Gross national income, converted to U.S. dollars using the World Bank Atlas method, divided by the midyear population	2017	https://data.worldbank.org/indicator/NY.GNP.PCAP.CD	182
Income inequality (Gini coefficient)	Measured income inequality, where the lowest possible value is zero and the highest being 100, when there is more concentration of wealth.	2016	http://wdi.worldbank.org/table/1.3#	157
Population living in urban areas (%)	The percentage of de facto population living in areas classified as urban according to the criteria used by each area or country as of 1 July of the year indicated.	2010	http://apps.who.int/gho/data/node.main.nURBPOP?lang=en	190
Gender Inequality index	Measures of gender disparity in the 3 dimensions of reproductive health, empowerment and labor market: the lowest	2017	http://www.hdr.undp.org/en/composite/GII	156

	possible score is 0 (equality) and highest possible score is 1 (inequality).			
Population of females with at least some secondary education (%)	Percentage of females aged ≥25 years who had at least some secondary education	2010-2017	http://www.hdr.undp.org/en/composite/GII	159
Poverty rate (%)	Percentage of population living below $1.25 a day (extreme poverty)	2007-2013	http://apps.who.int/gho/data/node.main.POP107?lang=en	115
Crude birth rate (per 1000 population)	Annual number of live births per 1,000 population.	2013	http://apps.who.int/gho/data/node.wrapper.imr?x-id=2978	190
**Lifestyle factors**				
Total alcohol per capita consumption 2016 (Litres of pure alcohol per person (15 years or older) per year)	Total (sum of recorded and unrecorded alcohol) amount of alcohol consumed per person (15years of age or older) over a calendar year, in liters of pure alcohol, adjusted for tourist consumption.	2016	http://apps.who.int/gho/data/node.main.SDG35?lang=en	189
Prevalence of current tobacco smoking among female, age-standardised (%)	The percentage of the population aged 15 years and over who currently use any tobacco product (smoked and/or smokeless tobacco) on a daily or non-daily basis.	2013	http://apps.who.int/gho/data/node.main.1250?lang=en	126
**Healthcare resources**				
Current health expenditure (CHE) as percentage of gross domestic product (GDP) (%)	Level of Current Health Expenditure expressed as a percentage of GDP	2015	http://apps.who.int/gho/data/view.main.GHEDCHEGDPSHA2011v?lang=en	190

Skilled health professionals density (per 10 000 population)	The total number of physicians, nursing and midwifery personnel per 10 000 population	2008-2016	http://apps.who.int/gho/data/node.main.HWF10?lang=en	178
**Maternal infections and conditions**				
Prevalence of anaemia in pregnant women (%)	Percentage of women aged 15−49 years with a haemoglobin concentration less than 110 g/L for pregnant women, adjusted for altitude and smoking.	2016	http://apps.who.int/gho/data/node.main.ANEMIA1?lang=en	186
Syphilis seropositivity among antenatal care attendees (%)	Percentage of antenatal care attendees who tested positive for syphilis.	2010-2017	http://apps.who.int/gho/data/node.main.A1359STI?lang=en	143
Prevalence of obesity among female adults (%)	Percentage of female adults (≥18 years) with a body mass index (BMI) of ≥30 kg/m^2. (age-standardized)	2016	http://apps.who.int/gho/data/node.main.A900A?lang=en	190
Prevalence of underweight among female adults (%)	Percentage of female adults (≥18 years) with a body mass index (BMI) of <18 kg/m^2. (age-standardized)	2016	http://apps.who.int/gho/data/view.main.NCDBMILT18Av	188
Prevalence of hypertension among female adults (≥18 years) (%)	Percent of defined population with raised blood pressure (systolic blood pressure ≥ 140 OR diastolic blood pressure ≥ 90).	2015	http://apps.who.int/gho/data/view.main.2464EST	190
Prevalence of diabetes mellitus among female adults (≥18 years) (%)	Percent of defined population with fasting glucose ≥126 mg/dl (7.0 mmol/l) or history of diagnosis with diabetes or use of	2014	http://apps.who.int/gho/data/view.main.2469	189

	insulin or oral hypoglycemic drugs.			
**Maternal and reproductive health service coverage**				
Antenatal care coverage - at least four visits (%)	Percentage of women aged 15-49 with a live birth in a given time period that received antenatal care four or more times.	2009-2016	http://apps.who.int/gho/data/node.main.ANTENATALCARECOVERAGE4?lang=en	148
Births attended by skilled health personnel (%)	Percentage of births attended by skilled health personnel (i.e. doctors, nurses or midwives)	2006-2016	http://apps.who.int/gho/data/node.main.SKILLEDBIRTHATTENDANTS?lang=en	182
Births by caesarean section (%)	Percentage of births by caesarean section among all live births in a given time period. Measured access to emergency care	2010-2016	http://apps.who.int/gho/data/view.main.BIRTHSBYCAESAREANv	171
Adolescent birth rate (per 1000 women aged 15-19 years)	The annual number of births to women aged 15-19 years per 1,000 women in that age group. It is also referred to as the age-specific fertility rate for women aged 15-19	2009-2016	http://apps.who.int/gho/data/node.main.REPADO39?lang=en	170
Child marriage (%)	Proportion of women aged 20-24 years who were married or in a union before age 18.	2010-2017	http://apps.who.int/gho/data/view.main.GSWCAH45v	122
Family planning needs satifisied (%)	Proportion of married or in-union women of reproductive age (aged 15-49 years) who have their need for family planning	2007-2016	http://apps.who.int/gho/data/view.main.GSWCAH30v	118

	satisfied with modern methods

Table S2: Bivariate spatial regression of the determinants of stillbirth

Variables	Spatial regression Coefficient	P-value	Model
GNI per capita, log	-5.49	<0.001	OLS
Income inquality (Gini coefficient)	0.38	0.0004	Spatial error
Urban residence	-0.23	<0.001	Spatial error
Current health expenditure	-0.70	0.001	Spatial error
Population of women with at least secondary education	-0.24	<0.001	OLS
Poverty rate, log	5.79	<0.001	OLS
Gender inequality	42.0	<0.001	Spatial error
Crude birth rate	0.76	<0.001	OLS
Total alcohol consumption	-1.16	<0.001	Spatial error
Tobacco consumption, log	-4.11	<0.001	Spatial error
Anaemia in pregnancy	0.62	<0.001	Spatial error
Syphilis seropositivity among pregnant women, log	2.40	<0.001	Spatial error
Obesity among female adults	-0.30	<0.001	Spatial error
Prevalance of underweight among female adults, log	8.57	<0.001	Spatial lag
Hypertension among female adults	1.20	<0.001	OLS
Diabetes mellitus among female adults	0.11	0.471	Spatial error
Density of skilled health personnel, log	-6.62	<0.001	Spatial error
ANC coverage	-0.35	<0.001	Spatial error
Skilled birth attendants during deliveries	-0.36	<0.001	Spatial lag
Adolescent birth rate, log	6.19	<0.001	Spatial error
Child marriage	0.35	<0.001	Spatial error
Deliveries by caesarean section	-0.46	<0.001	Spatial error
Family planning needs satisfied	-0.16	<0.001	Spatial lag

OLS- ordinary least square regression, GNI- Gross National Income

Table S3: Predicted scenarios of the inferential analysis based on joint probabilities of region and stillbirth

Region<br>All	Baseline (%)	Acceleration (%)
Stillbirth (Low rate)	56	100
Skilled attendants during delivery (High coverage)	70	88
ANC coverage (High coverage)	55	70
Anaemia in pregnancy (High prevalence)	27	11
Gender inequality (High index)	43	21
**Africa (AFR)**		
Stillbirth (Low rate)	14	100
Skilled attendants during delivery (High coverage)	32	67
ANC coverage (High coverage)	20	37
Anaemia in pregnancy (High prevalence)	61	31
Gender inequality (High index)	89	68
**South East Asia (SEAR)**		
Stillbirth (Low rate)	38	100
Skilled attendants during delivery (High coverage)	55	63
ANC coverage (High coverage)	62	67
Anaemia in pregnancy (High prevalence)	62	61
Gender inequality (High index)	69	47
**Eastern Mediterranean (EMR)**		
Stillbirth (Low rate)	50	100
Skilled attendants during delivery (High coverage)	54	71
ANC coverage (High coverage)	36	45
Anaemia in pregnancy (High prevalence)	33	26
Gender inequality (High index)	55	38
**Western Pacific (WPR)**		
Stillbirth (Low rate)	62	100
Skilled attendants during delivery (High coverage)	81	85
ANC coverage (High coverage)	47	56
Anaemia in pregnancy (High prevalence)	23	17
Gender inequality (High index)	29	23
**America (AMR)**		
Stillbirth (Low rate)	72	100
Skilled attendants during delivery (High coverage)	85	91
ANC coverage (High coverage)	85	85
Anaemia in pregnancy (High prevalence)	5	4
Gender inequality (High index)	41	31
**Europe (EUR)**		
Stillbirth (Low rate)	87	100
Skilled attendants during delivery (High coverage)	96	98
ANC coverage (High coverage)	77	78
Anaemia in pregnancy (High prevalence)	3	2
Gender inequality (High index)	1	1

SUPPLEMENTARY FIGURE

Figure S1: Sensitivity tornado plot for low stillbirth rate across WHO-regions, 2015

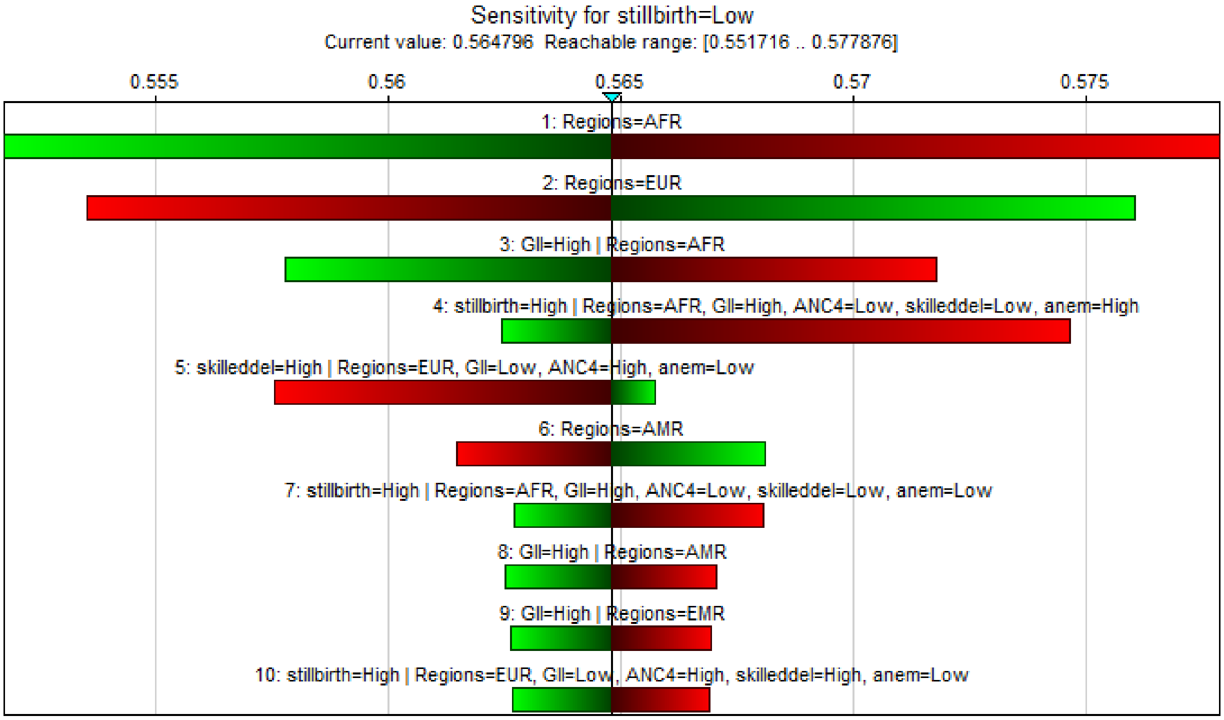

As seen in level 4 of the tornado plot, the factors that influenced high stillbirth rates were; African region, high gender inequality index, low ANC coverage, low skilled birth attendants during deliveries and high prevalence of anaemia in pregnancy.
